# Pharmacotherapies for stimulant use disorder and co-occurring attention deficit hyperactivity disorder: protocol for a systematic review and a meta-analysis

**DOI:** 10.3389/fpsyt.2025.1667614

**Published:** 2025-09-04

**Authors:** Henrique N. P. Oliva, Alejandra Pulido-Saavedra, Alisson Paredes-Naveda, Emerson Forselius, Marc N. Potenza, Oluwole O. Jegede, Gustavo A. Angarita

**Affiliations:** ^1^ Department of Psychiatry, Yale University School of Medicine, New Haven, CT, United States; ^2^ Connecticut Mental Health Center, New Haven, CT, United States; ^3^ Clinical Neuroscience Research Unit, Connecticut Mental Health Center, New Haven, CT, United States; ^4^ Southern Connecticut State University (SCSU), New Haven, CT, United States; ^5^ Syracuse University, Syracuse, NY, United States; ^6^ Child Study Center, Yale University School of Medicine, New Haven, CT, United States; ^7^ Department of Neuroscience, Yale University, New Haven, CT, United States; ^8^ Connecticut Council on Problem Gambling, Wethersfield, CT, United States; ^9^ Wu Tsai Institute, Yale University, New Haven, CT, United States

**Keywords:** stimulant use disorder, attention deficit hyperactivity disorder, cocaine, methamphetamine, co-occurring disorder

## Abstract

**Background:**

Stimulant use disorder (StUD) and attention-deficit/hyperactivity disorder (ADHD) frequently co-occur. This comorbidity complicates treatment and worsens clinical outcomes. Despite the high prevalence, shared vulnerability and clinical relevance of this comorbidity, evidence on effective pharmacotherapies among individuals with this dual diagnosis remains limited.

**Materials and methods:**

This systematic review protocol is reported in accordance with the Preferred Reporting Items for Systematic Reviews and Meta-Analyses Protocols (PRISMA-P) statement and will include randomized controlled trials involving adults with comorbid StUD (cocaine, amphetamines, or methamphetamines) and ADHD. The following databases will be searched: PubMed, Embase, Scopus, and Web of Science. *Covidence* will be used to support independent screening and data extraction. Two reviewers will independently screen studies (title/abstract and full text). One author will extract data, which will be independently verified by a second reviewer. Quality assessment of included articles will be assessed using the Cochrane Risk of Bias instrument, and certainty of the evidence for each outcome will be assessed using Grading of Recommendations, Assessment, Development and Evaluations (GRADE) methodology. Primary outcomes include duration of continuous abstinence, odds of stimulant-negative urine samples, ADHD symptom changes, and medication adverse events. Where feasible, meta-analyses will be conducted using random-effects models.

**Significance and dissemination:**

This review will synthesize existing evidence on the efficacy of pharmacotherapies (stimulants and non-stimulants) for individuals with co-occurring StUD and ADHD. The results of this study will likely inform clinical practice by evaluating outcomes such as reduction in stimulant use and abstinence, and improvement in ADHD symptoms. Findings will be disseminated through peer-reviewed publication and presentations to reach both clinical and academic audiences.

**Systematic review registration:**

PROSPERO, CRD420250655356.

## Introduction

Attention-deficit hyperactivity disorder (ADHD) frequently co-occurs with substance use disorders (SUDs), particularly stimulant use disorder (StUD) involving cocaine, methamphetamine, or prescription stimulants (1). The prevalence of ADHD among individuals with cocaine and methamphetamine use disorder is approximately 20% (1–3), markedly higher than the estimated 6.8% prevalence in the general adult population (4). Conversely, ADHD increases the risk of developing StUD, potentially due to overlapping neurobiological vulnerabilities and self-medication with stimulants (5). The comorbidity is linked to worse treatment outcomes, including reduced retention, lower abstinence rates, greater morbidity, and higher healthcare utilization (6–8).

Pharmacological options for ADHD include both stimulant (e.g., methylphenidate, amphetamines) and non-stimulant agents (e.g., atomoxetine, guanfacine, clonidine, viloxazine), alongside several off-label treatments such as bupropion and modafinil (9, 10). No medications are FDA-approved for StUD, but off-label agents – including bupropion, modafinil, disulfiram, and topiramate – are sometimes used (11, 12). The overlap in off-label agents for ADHD and StUD supports the hypothesis of shared pathophysiological mechanisms (7).

Evidence suggests that both disorders share alterations in dopaminergic, cholinergic, and GABAergic signaling (13–19), as well as structural and functional brain changes – particularly in prefrontal and anterior cingulate regions involved in executive control, reward processing, and impulse regulation (20–23). Such overlap may help explain anecdotal reports of therapeutic stimulant effects in individuals with ADHD and StUD (24–26) and the growing research interest in using stimulant medications for StUD (27).

While there is substantial literature on the treatment of ADHD and StUD individually, these studies may not fully capture the breadth of neurobiological alterations or the range of potentially effective pharmacological strategies when these conditions co-occur. Several randomized controlled trials (RCTs) have evaluated medications targeting either ADHD or StUD (28–30), and some of these have been synthesized in systematic reviews and meta-analyses (27). However, the field remains limited by a relative scarcity of double-blind RCTs specifically focused on dual-diagnosis populations. Existing reviews often aggregate findings from heterogeneous samples, making it difficult to draw definitive conclusions about efficacy or generalizability in individuals with co-occurring ADHD and StUD. As such, further targeted trials and meta-analytic efforts are needed to clarify treatment efficacy in this understudied group.

This protocol describes the methodology for a systematic review and meta-analysis of RCTs evaluating pharmacological treatments in adults with co-occurring ADHD and StUD. By synthesizing efficacy and safety data, the review aims to identify promising treatment strategies and inform future research.

## Methods

### Study registration

This protocol is reported in accordance with the Preferred Reporting Items for Systematic Reviews and Meta-Analyses Protocols (PRISMA-P) statement and registered in the PROSPERO database under the registration number CRD420250655356.

### Eligibility criteria

We will include RCTs that evaluate pharmacological interventions for adults (aged 18 years and older) with a dual diagnosis of StUD, including cocaine, methamphetamine, or prescription stimulant misuse, and ADHD. Eligible studies must have enrolled participants formally diagnosed with both conditions. Pharmacological interventions of interest will include stimulants (e.g., methylphenidate, amphetamines) and non-stimulants (e.g., atomoxetine, disulfiram, modafinil, topiramate).

A preliminary pilot search in PubMed yielded a small number of potentially eligible trials, confirming the feasibility of identifying studies meeting our criteria while also highlighting the scarcity of evidence in this specific comorbid population.

To be eligible, studies must have included individuals with co-occurring ADHD and StUD and reported at least one of the following outcomes: (1) treatment outcomes, including abstinence from stimulant use, measured via self-report, biologically verified abstinence (e.g., urine metabolites), and/or improvements in ADHD symptoms (e.g., hyperactivity, impulsivity, inattention) assessed through validated instruments such as the Adult ADHD Rating Scale (AARS), Clinical Global Impression (CGI), or Conners’ Adult ADHD Rating Scales (CAARS); and (2) safety outcomes, such as adverse events, side effects, or treatment tolerability. While other outcomes such as quality-of-life measures (e.g., World Health Organization Quality of Life Brief Version [WHOQOL-BREF] or 36-Item Short Form Health Survey [SF-36]) would provide valuable insights into functional outcomes, our preliminary search identified no trials reporting such data for StUD populations in this dual-diagnosis context. Should any eligible studies emerge during full screening that include quality of life measures, we will document and analyze these findings descriptively. No language restrictions will be applied.

We will exclude studies involving participants with primary diagnoses of SUDs other than StUD and studies that do not report outcomes based on pharmacotherapy treatments in individuals with co-occurring ADHD and StUD. Additional exclusion criteria will include animal studies, qualitative research, reviews, case reports, conference abstracts, and proceedings.

### Collection and analysis of the data

#### Information sources

We will search PubMed, Embase, Scopus, Web of Science, and CINAHL (via the EBSCOhost platform) to identify relevant studies. Additionally, we will search ClinicalTrials.gov to capture unpublished and ongoing trials. To ensure comprehensive coverage, we will also manually screen the reference lists of included studies and relevant systematic reviews or meta-analyses to identify additional eligible publications not captured through the primary searches.

#### Search strategy

The search strategy was developed using a combination of relevant keywords and medical subject headings related to stimulant use disorder, attention-deficit hyperactivity disorder, comorbidity, pharmacotherapy, and specific medications of interest. Filters will be applied for clinical trials as the publication type. The strategy will be adapted for each database to ensure appropriate syntax and indexing. The complete search syntax for PubMed and Scopus are provided in the **Supplementary Table 1**.

#### Study selection process

Two independent reviewers will screen all titles and abstracts identified by the search. Studies meeting the inclusion criteria will undergo full-text review by two authors. Disagreements between reviewers will be resolved through discussion or by involving a third reviewer. The inclusion and exclusion process will be documented, with reasons for exclusion noted.

#### Data extraction

Data will be extracted by two independent reviewers using a standardized data extraction form. Extracted information will include:

- Study Characteristics: author(s), publication year, country, study design, sample size, participant demographics.
- Interventions: type of pharmacotherapy (e.g., medication name, dosage, administration).
- Outcomes: measures of efficacy (e.g., abstinence from stimulants, improvements in ADHD symptoms), and other relevant secondary outcomes such as safety (e.g., adverse events).

#### Quality assessment

The risk of bias of the included studies will be assessed using the Cochrane risk of bias tool for randomized trials (RoB2) (31). Each study will be evaluated across several domains, including selection bias, performance bias, detection bias, and reporting bias. We also assess publication bias using funnel plots and Egger’s test if ≥10 studies are available. Any potential sources of bias will be documented.

The quality of evidence for all outcomes will be evaluated using the Grading of Recommendations, Assessment, Development and Evaluations (GRADE) methodology. This systematic approach examines five key domains: risk of bias, precision, directness, consistency across studies, and potential publication bias to determine the overall confidence in the estimated effects (32).

### Outcomes

For substance use, primary outcomes will include (1) abstinence duration (measured as longest continuous stimulant-free period via biologically verified methods like urine toxicology, to be pooled as standardized mean differences [SMDs]) and (2) odds of stimulant-negative urine samples during treatment (pooled as odds ratios [ORs]).

For ADHD, primary outcomes will comprise (1) symptom severity (measured by validated scales such as the AARS or CAARS, pooled as SMDs) and (2) clinical improvement (proportion of participants rating improvement on the CGI scale, pooled as ORs).

Secondary outcomes will focus on safety, including treatment-emergent adverse events and withdrawal rates. The comparisons will be performed between active treatment vs. placebo within each drug group. If feasible, depending on the number of included studies, we will also compare efficacy across drug classes (stimulants vs. non-stimulants). Continuous outcomes will be analyzed as SMDs with 95% CIs and dichotomous outcomes as ORs with 95% CIs. Where operational definitions diverge (e.g., abstinence criteria), separate analyses will be conducted if sufficient data are available.

### Data synthesis

Data from eligible studies will be synthesized qualitatively when appropriate. In addition, a narrative description will summarize study characteristics, medication types, and outcome measures for all included trials, providing context for the quantitative findings. We will summarize intervention characteristics, participant populations, outcome measures, and direction and magnitude of effects, highlighting consistencies and discrepancies across studies. Where applicable, findings will be organized into thematic domains (e.g., treatment outcome, safety) and presented in a summary table to aid comparison. If studies are sufficiently homogeneous, a meta-analysis will be performed using fixed or random-effects models, as appropriate, to estimate pooled effect sizes for the primary outcomes. Heterogeneity will be assessed using the I² statistic. Because the included pharmacotherapies may vary in mechanism of action, we will address this potential source of heterogeneity by conducting subgroup analyses stratified by medication class (e.g., stimulants vs. non-stimulants) and, where data permit, by individual agents. Additionally, we plan to conduct leave-one-out analyses and analyses excluding high-risk-of-bias studies to evaluate the robustness of pooled estimates and identify whether individual studies exert a disproportionate influence on the overall findings. To address the biological and clinical relevance of effect sizes, we will interpret pooled results in the context of established minimally important differences (when available) and recognized benchmarks for meaningful change in stimulant use and ADHD symptomatology. Where such thresholds are not well established, we will consider the magnitude of observed changes alongside their potential impact on functional outcomes, safety, and patient-centered measures, thereby ensuring that statistical significance is evaluated within a clinically meaningful framework. The completed PRISMA-P table is available in **Supplementary Table 2**.

## Discussion

This protocol outlines the methodology for a systematic review and meta-analysis examining pharmacological treatments for individuals with co-occurring ADHD and StUD. Designed in accordance with PRISMA-P guidelines, the protocol aims to employ a comprehensive search strategy, clearly defined eligibility criteria, and standardized data extraction and quality assessment tools to ensure rigor and reproducibility. By focusing on RCTs, this study seeks to provide high-quality evidence in an area where clinical decision-making remains complex.

This study protocol is not without potential limitations. The exclusion of non-RCT studies may limit insights into real-world treatment effects, and high attrition rates in existing trials may reduce generalizability. Despite this constraint, the proposed review will address a significant gap in the literature by synthesizing data specific to populations with co-occurring ADHD and StUD.

While pharmacological interventions alone may have limited efficacy on substance use, their integration with targeted psychosocial treatments is widely recommended for optimal care; however, the study’s focus on pharmacotherapies will exclude psychosocial treatments (27). Notwithstanding these limitations, this protocol builds on existing critical evidence-based work to generate a review aimed to guide future research and inform best practices for treating individuals with co-occurring ADHD and StUD.

## Author’s note

This publication does not express the views of the Department of Mental Health and Addiction Services or the State of Connecticut. The views and opinions expressed are those of the authors.
